# 6,6′-Dimeth­oxy-2,2′-[(*E*,*E*′)-(2,4,6-trimethyl-1,3-phenyl­ene)bis­(nitrilo­methanylyl­idene)]diphenol chloro­form monosolvate

**DOI:** 10.1107/S1600536812011531

**Published:** 2012-03-24

**Authors:** Hadariah Bahron, Najihah Abu Bakar, Bohari M. Yamin, M. Sukeri M. Yusof

**Affiliations:** a Department of Chemistry, Faculty of Applied Sciences, Universiti Teknologi MARA, 40450 Shah Alam, Selangor, Malaysia; b Department of Chemical Sciences and Food Technology, Faculty of Science and Technology, Universiti Kebangsaan Malaysia, 43650 Bangi, Selangor, Malaysia; c Department of Chemical Sciences, Faculty of Science and Technology, Universiti Malaysia Terengganu, 21030 Kuala Terengganu, Terengganu, Malaysia

## Abstract

In the title compound, C_25_H_26_N_2_O_4_·CHCl_3_, the aromatic rings of the imino­methyl-6-meth­oxy­phenol fragments make dihedral angles of 58.33 (6) and 87.74 (6)° with the central benzene ring. The mol­ecular conformation is stabilized by intra­molecular O—H⋯N hydrogen bonds. In the crystal, an inter­molecular C—H⋯O hydrogen bond involving the chloro­form solvent mol­ecule is observed. The crystal packing is further stabilized by π–π stacking inter­actions [centroid–centroid distances = 3.739 (3)–3.776 (3) Å] between the benzene rings of centrosymmetrically related mol­ecules.

## Related literature

For a related structure, see: Yamin *et al.* (2009 ▶). For the synthetic procedure, see: Hernández-Molina *et al.* (1997 ▶). For standard bond lengths, see: Allen *et al.* (1987 ▶). 
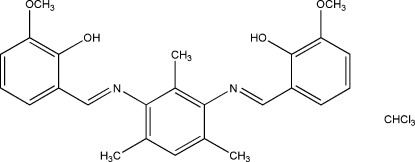


## Experimental

### 

#### Crystal data

C_25_H_26_N_2_O_4_·CHCl_3_
*M* *_r_* = 537.85
Triclinic, 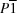

*a* = 10.162 (2) Å
*b* = 10.486 (2) Å
*c* = 12.640 (3) Å
α = 99.315 (4)°
β = 93.140 (4)°
γ = 90.196 (4)°
*V* = 1327.0 (5) Å^3^
*Z* = 2
Mo *K*α radiation
μ = 0.38 mm^−1^
*T* = 298 K
0.50 × 0.47 × 0.44 mm

#### Data collection

Bruker SMART APEX CCD area-detector diffractometer
Absorption correction: multi-scan (*SADABS*; Bruker, 2000 ▶) *T*_min_ = 0.833, *T*_max_ = 0.851
14608 measured reflections
4947 independent reflections
3799 reflections with *I* > 2σ(*I*)
*R*_int_ = 0.017

#### Refinement

*R*[*F* ^2^ > 2σ(*F* ^2^)] = 0.051
*wR*(*F* ^2^) = 0.150
*S* = 1.04
4947 reflections
329 parameters
H atoms treated by a mixture of independent and constrained refinement
Δρ_max_ = 0.42 e Å^−3^
Δρ_min_ = −0.43 e Å^−3^

### 

Data collection: *SMART* (Bruker, 2000 ▶); cell refinement: *SAINT* (Bruker, 2000 ▶); data reduction: *SAINT*; program(s) used to solve structure: *SHELXTL* (Sheldrick, 2008 ▶); program(s) used to refine structure: *SHELXTL*; molecular graphics: *SHELXTL*; software used to prepare material for publication: *SHELXTL*, *PARST* (Nardelli, 1995 ▶) and *PLATON* (Spek, 2009 ▶).

## Supplementary Material

Crystal structure: contains datablock(s) global, I. DOI: 10.1107/S1600536812011531/rz2720sup1.cif

Structure factors: contains datablock(s) I. DOI: 10.1107/S1600536812011531/rz2720Isup2.hkl

Additional supplementary materials:  crystallographic information; 3D view; checkCIF report

## Figures and Tables

**Table 1:** Hydrogen-bond geometry (Å, °)

*D*—H⋯*A*	*D*—H	H⋯*A*	*D*⋯*A*	*D*—H⋯*A*
O1—H1⋯N1	0.89 (3)	1.76 (4)	2.566 (3)	150 (3)
O3—H3⋯N2	0.87 (4)	1.83 (4)	2.618 (2)	150 (4)
C26—H26⋯O1^i^	0.98	2.16	3.071 (4)	154

Symmetry code: (i) 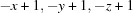.

**Fig. 1.**
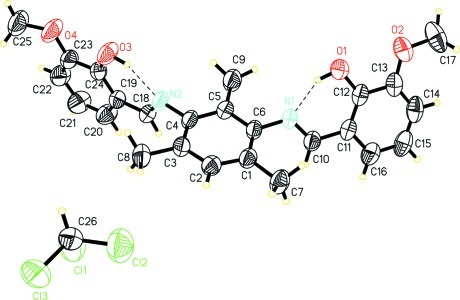
The molecular structure of the title compound, with displacement ellipsods drawn at the 50% probability level. Intramolecular hydrogen bonds are shown as dashed lines.

**Fig. 2.**
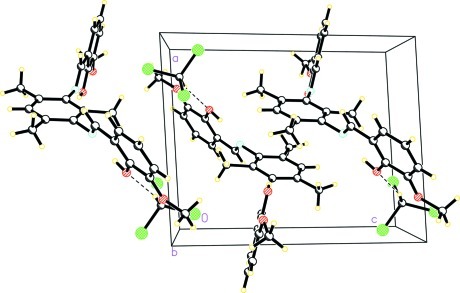
A packing diagram of the title compound approximately viewed down the b axis. Intermolecular hydrogen bonds are shown as dashed lines.

## supplementary crystallographic information

### Comment

The title compound is analogous to the previously reported compound 6,6'-Dimethoxy-2,2'-[(E,E')-(4-chloro-m-phenylene)bis(nitrilomethylidyne)]diphenol (Yamin *et al.*, 2009) except for the presence of three methyl groups at positions 2, 4 and 6 and a solvate chloroform molecule (Fig. 1). The molecule exhibits a butterfly-like shape. The bond lengths are in the normal ranges (Allen *et al.*, 1987) and comparable with those reported for similar molecules. One 2-iminomethyl-6-methoxyphenol wing (N1/C11—C17/O1/O2) is planar with a maximum deviation of 0.091 (4) Å for atom C17. The other wing (N2/C18—C25/O3/O4) is slightly twisted, with atom C25 deviating by 0.304 (4) Å. The central benzene ring (C1—C9) makes dihedral angle of 58.33 (6) and 87.74 (6) Å with N1/C11—C17/O1/O2 and O3/O4/C19—C24 wings, respectively. The dihedral angle between the two wings is 58.31 (10)°. There are two intramolecular O—H···N hydrogen bonds (Table 1) stabilizing the molecular conformation. In the crystal structure, weak intemolecular C—H···O hydrogen bonds are observed (Table 1) involving the chloroform solvated molecule (Fig. 2). The crystal packing is further stabilized by π–π stacking interactions occurring between centrosymmetrically-related molecules (Cg1···Cg1^i^ = 3.776 (3) Å; Cg2···Cg2^ii^ = 3.739 (3) Å; Cg1 and Cg2 are the centroids of the C19–C24 and C1–C6 rings, respectively (symmetry codes: (i) 1-x, -y, -z; (ii) 1-x, 1-y, 1-z).

### Experimental

The Schiff base was synthesized by refluxing in ethanol 2,4,6-trimetyhl-1,3-phenylenediamine (0.4507 g, 3.0 mmol) and (*o*-vanillin (0.9998 g,6.0mmol) for 5 h as previously described by Hernández-Molina *et al.* (1997). The solvent was then evaporated off under reduced pressure. The viscous solution obtained was left in room conditions for a week affording a solid product which was recrystallized from chloroform. The yellow single crystals obtained were suitable for X-ray crystallographic investigation. Yield 92%. Melting point: 417–421 K. Analytical calculation for C_25_H_26_N_2_O_4_ [3CH_3_-mpd(*o* -van)_2_]: C, 71.75; H, 6.26; N, 6.69. Found: C, 71.90; H, 6.42; N, 6.84. IR (cm^-1^): ν(C=N) 1611.7 (*m*), ν(C–O–C) 1253.9 (*s*), ν(C–OH) 1212.7 (*w*), ν(C–Cl) 1099.8 (*w*). ^1^H NMR (CDCl_3_, 300 MHz, p.p.m.): δ = 13.5027 (2*H*, *s*, OH), 8.364 (2*H*, *s*, HC=N), 7.061–6.898 (7*H*, *m*, *H*-Aryl), 3.975 (6*H*, *s*, OCH_3_), 2.208 (6*H*, *s*, CH_3_), 2.071 (3*H*, *s*, CH_3_).

### Refinement

H atoms on C were positioned geometrically with C—H = 0.93–0.96 Å, and constrained to ride on their parent atoms with *U*_iso_(H) = *xU*_eq_(C) where *x* = 1.5 for methyl H and *x* = 1.2 for aromatic H atoms. The hydroxy H atoms were located from a Fourier difference map and refined isotropically.

### Crystal data

**Table:**

C_25_H_26_N_2_O_4_·CHCl_3_	*Z* = 2
*M**_r_* = 537.85	*F*(000) = 560
Triclinic, *P*1	*D*_x_ = 1.346 Mg m^−^^3^
Hall symbol: -P 1	Mo *K*α radiation, λ = 0.71073 Å
*a* = 10.162 (2) Å	Cell parameters from 5967 reflections
*b* = 10.486 (2) Å	θ = 2.0–25.5°
*c* = 12.640 (3) Å	µ = 0.38 mm^−^^1^
α = 99.315 (4)°	*T* = 298 K
β = 93.140 (4)°	Block, colourless
γ = 90.196 (4)°	0.50 × 0.47 × 0.44 mm
*V* = 1327.0 (5) Å^3^	

### Data collection

**Table:**

Bruker SMART APEX CCD area-detector diffractometer	4947 independent reflections
Radiation source: fine-focus sealed tube	3799 reflections with *I* > 2σ(*I*)
Graphite monochromator	*R*_int_ = 0.017
Detector resolution: 83.66 pixels mm^-1^	θ_max_ = 25.5°, θ_min_ = 2.0°
ω scan	*h* = −12→12
Absorption correction: multi-scan (*SADABS*; Bruker, 2000)	*k* = −12→12
*T*_min_ = 0.833, *T*_max_ = 0.851	*l* = −15→15
14608 measured reflections	

### Refinement

**Table:**

Refinement on *F*^2^	Primary atom site location: structure-invariant direct methods
Least-squares matrix: full	Secondary atom site location: difference Fourier map
*R*[*F*^2^ > 2σ(*F*^2^)] = 0.051	Hydrogen site location: inferred from neighbouring sites
*wR*(*F*^2^) = 0.150	H atoms treated by a mixture of independent and constrained refinement
*S* = 1.04	*w* = 1/[σ^2^(*F*_o_^2^) + (0.0709*P*)^2^ + 0.542*P*] where *P* = (*F*_o_^2^ + 2*F*_c_^2^)/3
4947 reflections	(Δ/σ)_max_ < 0.001
329 parameters	Δρ_max_ = 0.42 e Å^−^^3^
0 restraints	Δρ_min_ = −0.43 e Å^−^^3^

### Special details

**Table:**

Geometry. All e.s.d.'s (except the e.s.d. in the dihedral angle between two l.s. planes) are estimated using the full covariance matrix. The cell e.s.d.'s are taken into account individually in the estimation of e.s.d.'s in distances, angles and torsion angles; correlations between e.s.d.'s in cell parameters are only used when they are defined by crystal symmetry. An approximate (isotropic) treatment of cell e.s.d.'s is used for estimating e.s.d.'s involving l.s. planes.
Refinement. Refinement of *F*^2^ against ALL reflections. The weighted *R*-factor *wR* and goodness of fit *S* are based on *F*^2^, conventional *R*-factors *R* are based on *F*, with *F* set to zero for negative *F*^2^. The threshold expression of *F*^2^ > σ(*F*^2^) is used only for calculating *R*-factors(gt) *etc*. and is not relevant to the choice of reflections for refinement. *R*-factors based on *F*^2^ are statistically about twice as large as those based on *F*, and *R*- factors based on ALL data will be even larger.

### Fractional atomic coordinates and isotropic or equivalent isotropic displacement parameters (Å^2^)

**Table:**

	*x*	*y*	*z*	*U*_iso_*/*U*_eq_	
Cl1	0.03110 (10)	0.62554 (11)	0.86722 (8)	0.1141 (4)	
Cl2	0.27407 (11)	0.52065 (10)	0.93526 (9)	0.1134 (3)	
Cl3	0.12916 (12)	0.69304 (10)	1.08602 (7)	0.1112 (4)	
O1	0.66016 (19)	0.17533 (16)	0.16568 (15)	0.0666 (5)	
O2	0.78095 (19)	0.01822 (19)	0.02179 (15)	0.0774 (5)	
O3	0.24675 (16)	0.87363 (16)	0.38440 (18)	0.0742 (6)	
O4	0.12149 (18)	1.08231 (16)	0.3545 (2)	0.0897 (7)	
N1	0.47101 (18)	0.21937 (16)	0.29251 (15)	0.0518 (4)	
N2	0.24295 (18)	0.62506 (16)	0.38954 (16)	0.0527 (4)	
C1	0.4031 (2)	0.28405 (19)	0.47697 (18)	0.0500 (5)	
C2	0.3505 (2)	0.3788 (2)	0.55115 (18)	0.0536 (5)	
H2	0.3515	0.3665	0.6224	0.064*	
C3	0.2966 (2)	0.4910 (2)	0.52441 (18)	0.0512 (5)	
C4	0.2981 (2)	0.50847 (18)	0.41760 (18)	0.0473 (5)	
C5	0.3573 (2)	0.42049 (19)	0.34071 (17)	0.0492 (5)	
C6	0.4070 (2)	0.30635 (19)	0.37139 (18)	0.0474 (5)	
C7	0.4551 (3)	0.1628 (2)	0.5135 (2)	0.0662 (6)	
H7A	0.3897	0.0952	0.4967	0.099*	
H7B	0.4750	0.1794	0.5896	0.099*	
H7C	0.5336	0.1367	0.4774	0.099*	
C8	0.2394 (3)	0.5905 (2)	0.6084 (2)	0.0705 (7)	
H8A	0.1449	0.5873	0.5993	0.106*	
H8B	0.2707	0.6749	0.6010	0.106*	
H8C	0.2660	0.5726	0.6785	0.106*	
C9	0.3669 (3)	0.4462 (2)	0.2278 (2)	0.0721 (7)	
H9A	0.2897	0.4123	0.1851	0.108*	
H9B	0.4437	0.4050	0.1977	0.108*	
H9C	0.3735	0.5376	0.2283	0.108*	
C10	0.4370 (2)	0.1001 (2)	0.26946 (18)	0.0520 (5)	
H10	0.3665	0.0698	0.3027	0.062*	
C11	0.5068 (2)	0.0109 (2)	0.19195 (18)	0.0515 (5)	
C12	0.6140 (2)	0.0524 (2)	0.14253 (18)	0.0529 (5)	
C13	0.6780 (2)	−0.0339 (2)	0.06563 (19)	0.0599 (6)	
C14	0.6338 (3)	−0.1596 (2)	0.0417 (2)	0.0716 (7)	
H14	0.6758	−0.2175	−0.0089	0.086*	
C15	0.5280 (3)	−0.2015 (2)	0.0915 (2)	0.0767 (8)	
H15	0.4998	−0.2872	0.0745	0.092*	
C16	0.4642 (3)	−0.1179 (2)	0.1656 (2)	0.0655 (6)	
H16	0.3927	−0.1467	0.1985	0.079*	
C17	0.8454 (3)	−0.0624 (4)	−0.0602 (3)	0.1006 (11)	
H17A	0.8850	−0.1334	−0.0315	0.151*	
H17B	0.9125	−0.0134	−0.0870	0.151*	
H17C	0.7824	−0.0949	−0.1177	0.151*	
C18	0.1223 (2)	0.6255 (2)	0.36095 (18)	0.0513 (5)	
H18	0.0749	0.5483	0.3533	0.062*	
C19	0.0532 (2)	0.7410 (2)	0.33923 (17)	0.0498 (5)	
C20	−0.0803 (2)	0.7342 (3)	0.3063 (2)	0.0689 (7)	
H20	−0.1244	0.6549	0.2957	0.083*	
C21	−0.1466 (3)	0.8424 (3)	0.2894 (3)	0.0801 (8)	
H21	−0.2353	0.8364	0.2666	0.096*	
C22	−0.0827 (3)	0.9608 (3)	0.3060 (2)	0.0720 (7)	
H22	−0.1290	1.0346	0.2956	0.086*	
C23	0.0486 (2)	0.9705 (2)	0.3378 (2)	0.0595 (6)	
C24	0.1182 (2)	0.8603 (2)	0.35432 (18)	0.0499 (5)	
C25	0.0571 (3)	1.1989 (3)	0.3692 (3)	0.0928 (10)	
H25A	0.0083	1.2092	0.3040	0.139*	
H25B	0.1205	1.2681	0.3880	0.139*	
H25C	−0.0023	1.2005	0.4259	0.139*	
C26	0.1719 (3)	0.6559 (3)	0.9533 (2)	0.0741 (7)	
H26	0.2196	0.7299	0.9349	0.089*	
H3	0.275 (3)	0.797 (4)	0.392 (3)	0.109 (11)*	
H1	0.605 (3)	0.218 (3)	0.210 (3)	0.101 (11)*	

### Atomic displacement parameters (Å^2^)

**Table:**

	*U*^11^	*U*^22^	*U*^33^	*U*^12^	*U*^13^	*U*^23^
Cl1	0.1134 (7)	0.1233 (8)	0.0967 (6)	0.0259 (6)	−0.0080 (5)	−0.0049 (5)
Cl2	0.1231 (8)	0.1002 (7)	0.1177 (7)	0.0351 (6)	0.0131 (6)	0.0177 (5)
Cl3	0.1584 (9)	0.1091 (7)	0.0672 (5)	−0.0003 (6)	0.0309 (5)	0.0097 (4)
O1	0.0770 (12)	0.0468 (9)	0.0746 (11)	0.0004 (8)	0.0250 (9)	−0.0016 (8)
O2	0.0793 (12)	0.0798 (12)	0.0697 (11)	0.0162 (10)	0.0216 (9)	−0.0039 (9)
O3	0.0490 (9)	0.0427 (9)	0.1321 (17)	0.0056 (7)	−0.0044 (9)	0.0210 (10)
O4	0.0635 (11)	0.0417 (9)	0.167 (2)	0.0105 (8)	0.0093 (12)	0.0248 (11)
N1	0.0546 (10)	0.0388 (9)	0.0620 (11)	0.0110 (8)	0.0091 (8)	0.0065 (8)
N2	0.0534 (11)	0.0344 (9)	0.0710 (12)	0.0073 (7)	0.0058 (9)	0.0095 (8)
C1	0.0467 (11)	0.0418 (11)	0.0622 (13)	0.0061 (9)	0.0012 (10)	0.0108 (9)
C2	0.0590 (13)	0.0491 (12)	0.0537 (12)	0.0043 (10)	0.0039 (10)	0.0112 (10)
C3	0.0528 (12)	0.0405 (11)	0.0595 (13)	0.0037 (9)	0.0080 (10)	0.0035 (9)
C4	0.0465 (11)	0.0322 (10)	0.0632 (13)	0.0035 (8)	0.0039 (9)	0.0075 (9)
C5	0.0522 (12)	0.0391 (10)	0.0570 (12)	0.0042 (9)	0.0063 (9)	0.0086 (9)
C6	0.0442 (11)	0.0361 (10)	0.0611 (13)	0.0061 (8)	0.0058 (9)	0.0048 (9)
C7	0.0717 (16)	0.0554 (14)	0.0740 (16)	0.0197 (12)	−0.0027 (12)	0.0193 (12)
C8	0.0881 (18)	0.0522 (14)	0.0700 (16)	0.0146 (12)	0.0194 (14)	0.0012 (12)
C9	0.099 (2)	0.0565 (14)	0.0644 (15)	0.0228 (13)	0.0172 (14)	0.0164 (12)
C10	0.0503 (12)	0.0442 (12)	0.0611 (13)	0.0078 (9)	0.0026 (10)	0.0078 (10)
C11	0.0571 (13)	0.0389 (11)	0.0565 (12)	0.0099 (9)	−0.0030 (10)	0.0041 (9)
C12	0.0609 (13)	0.0429 (11)	0.0531 (12)	0.0129 (10)	−0.0018 (10)	0.0043 (9)
C13	0.0659 (15)	0.0569 (14)	0.0540 (13)	0.0201 (11)	−0.0001 (11)	0.0014 (10)
C14	0.0876 (19)	0.0573 (15)	0.0625 (15)	0.0236 (13)	−0.0011 (14)	−0.0115 (12)
C15	0.095 (2)	0.0425 (13)	0.0849 (19)	0.0075 (13)	−0.0076 (16)	−0.0082 (12)
C16	0.0721 (16)	0.0461 (13)	0.0753 (16)	0.0021 (11)	−0.0032 (13)	0.0034 (11)
C17	0.082 (2)	0.125 (3)	0.082 (2)	0.0240 (19)	0.0192 (16)	−0.0254 (19)
C18	0.0572 (13)	0.0373 (10)	0.0595 (13)	0.0008 (9)	0.0072 (10)	0.0066 (9)
C19	0.0520 (12)	0.0442 (11)	0.0544 (12)	0.0071 (9)	0.0059 (9)	0.0102 (9)
C20	0.0565 (14)	0.0592 (14)	0.0923 (19)	−0.0024 (11)	−0.0047 (13)	0.0196 (13)
C21	0.0532 (14)	0.0771 (18)	0.113 (2)	0.0059 (13)	−0.0115 (14)	0.0308 (16)
C22	0.0593 (15)	0.0619 (15)	0.101 (2)	0.0180 (12)	0.0056 (14)	0.0310 (14)
C23	0.0548 (13)	0.0452 (12)	0.0817 (16)	0.0100 (10)	0.0116 (11)	0.0177 (11)
C24	0.0452 (11)	0.0457 (11)	0.0603 (13)	0.0090 (9)	0.0083 (9)	0.0109 (9)
C25	0.086 (2)	0.0506 (15)	0.141 (3)	0.0160 (14)	0.0102 (19)	0.0142 (17)
C26	0.0911 (19)	0.0671 (16)	0.0665 (16)	−0.0008 (14)	0.0179 (14)	0.0136 (13)

### Geometric parameters (Å, º)

**Table:**

Cl1—C26	1.745 (3)	C9—H9B	0.9600
Cl2—C26	1.753 (3)	C9—H9C	0.9600
Cl3—C26	1.738 (3)	C10—C11	1.457 (3)
O1—C12	1.351 (3)	C10—H10	0.9300
O1—H1	0.89 (4)	C11—C12	1.388 (3)
O2—C13	1.363 (3)	C11—C16	1.399 (3)
O2—C17	1.419 (3)	C12—C13	1.406 (3)
O3—C24	1.340 (3)	C13—C14	1.372 (4)
O3—H3	0.88 (4)	C14—C15	1.380 (4)
O4—C23	1.366 (3)	C14—H14	0.9300
O4—C25	1.379 (3)	C15—C16	1.368 (4)
N1—C10	1.280 (3)	C15—H15	0.9300
N1—C6	1.425 (3)	C16—H16	0.9300
N2—C18	1.259 (3)	C17—H17A	0.9600
N2—C4	1.435 (3)	C17—H17B	0.9600
C1—C2	1.381 (3)	C17—H17C	0.9600
C1—C6	1.394 (3)	C18—C19	1.458 (3)
C1—C7	1.509 (3)	C18—H18	0.9300
C2—C3	1.383 (3)	C19—C24	1.394 (3)
C2—H2	0.9300	C19—C20	1.395 (3)
C3—C4	1.392 (3)	C20—C21	1.362 (4)
C3—C8	1.505 (3)	C20—H20	0.9300
C4—C5	1.391 (3)	C21—C22	1.380 (4)
C5—C6	1.404 (3)	C21—H21	0.9300
C5—C9	1.503 (3)	C22—C23	1.370 (3)
C7—H7A	0.9600	C22—H22	0.9300
C7—H7B	0.9600	C23—C24	1.396 (3)
C7—H7C	0.9600	C25—H25A	0.9600
C8—H8A	0.9600	C25—H25B	0.9600
C8—H8B	0.9600	C25—H25C	0.9600
C8—H8C	0.9600	C26—H26	0.9800
C9—H9A	0.9600		
			
C12—O1—H1	106 (2)	O2—C13—C14	126.2 (2)
C13—O2—C17	117.6 (2)	O2—C13—C12	114.9 (2)
C24—O3—H3	107 (2)	C14—C13—C12	118.9 (2)
C23—O4—C25	118.9 (2)	C13—C14—C15	121.0 (2)
C10—N1—C6	121.59 (19)	C13—C14—H14	119.5
C18—N2—C4	118.54 (18)	C15—C14—H14	119.5
C2—C1—C6	117.92 (19)	C16—C15—C14	120.5 (2)
C2—C1—C7	119.0 (2)	C16—C15—H15	119.7
C6—C1—C7	123.0 (2)	C14—C15—H15	119.7
C1—C2—C3	123.1 (2)	C15—C16—C11	119.9 (3)
C1—C2—H2	118.5	C15—C16—H16	120.0
C3—C2—H2	118.5	C11—C16—H16	120.0
C2—C3—C4	117.53 (19)	O2—C17—H17A	109.5
C2—C3—C8	120.8 (2)	O2—C17—H17B	109.5
C4—C3—C8	121.6 (2)	H17A—C17—H17B	109.5
C5—C4—C3	121.95 (18)	O2—C17—H17C	109.5
C5—C4—N2	120.19 (19)	H17A—C17—H17C	109.5
C3—C4—N2	117.76 (18)	H17B—C17—H17C	109.5
C4—C5—C6	118.1 (2)	N2—C18—C19	123.36 (19)
C4—C5—C9	121.06 (19)	N2—C18—H18	118.3
C6—C5—C9	120.87 (19)	C19—C18—H18	118.3
C1—C6—C5	121.22 (19)	C24—C19—C20	119.0 (2)
C1—C6—N1	121.34 (18)	C24—C19—C18	120.59 (19)
C5—C6—N1	117.24 (19)	C20—C19—C18	120.4 (2)
C1—C7—H7A	109.5	C21—C20—C19	120.7 (2)
C1—C7—H7B	109.5	C21—C20—H20	119.7
H7A—C7—H7B	109.5	C19—C20—H20	119.7
C1—C7—H7C	109.5	C20—C21—C22	120.3 (2)
H7A—C7—H7C	109.5	C20—C21—H21	119.9
H7B—C7—H7C	109.5	C22—C21—H21	119.9
C3—C8—H8A	109.5	C23—C22—C21	120.4 (2)
C3—C8—H8B	109.5	C23—C22—H22	119.8
H8A—C8—H8B	109.5	C21—C22—H22	119.8
C3—C8—H8C	109.5	O4—C23—C22	125.1 (2)
H8A—C8—H8C	109.5	O4—C23—C24	114.9 (2)
H8B—C8—H8C	109.5	C22—C23—C24	120.0 (2)
C5—C9—H9A	109.5	O3—C24—C19	122.20 (18)
C5—C9—H9B	109.5	O3—C24—C23	118.2 (2)
H9A—C9—H9B	109.5	C19—C24—C23	119.6 (2)
C5—C9—H9C	109.5	O4—C25—H25A	109.5
H9A—C9—H9C	109.5	O4—C25—H25B	109.5
H9B—C9—H9C	109.5	H25A—C25—H25B	109.5
N1—C10—C11	121.1 (2)	O4—C25—H25C	109.5
N1—C10—H10	119.5	H25A—C25—H25C	109.5
C11—C10—H10	119.5	H25B—C25—H25C	109.5
C12—C11—C16	119.4 (2)	Cl3—C26—Cl1	110.58 (17)
C12—C11—C10	120.85 (19)	Cl3—C26—Cl2	110.98 (16)
C16—C11—C10	119.7 (2)	Cl1—C26—Cl2	109.26 (16)
O1—C12—C11	121.96 (19)	Cl3—C26—H26	108.7
O1—C12—C13	117.9 (2)	Cl1—C26—H26	108.7
C11—C12—C13	120.2 (2)	Cl2—C26—H26	108.7
			
C6—C1—C2—C3	3.1 (3)	C17—O2—C13—C14	3.3 (4)
C7—C1—C2—C3	−177.8 (2)	C17—O2—C13—C12	−176.9 (2)
C1—C2—C3—C4	−1.0 (3)	O1—C12—C13—O2	−0.5 (3)
C1—C2—C3—C8	179.3 (2)	C11—C12—C13—O2	179.3 (2)
C2—C3—C4—C5	−3.3 (3)	O1—C12—C13—C14	179.3 (2)
C8—C3—C4—C5	176.4 (2)	C11—C12—C13—C14	−0.9 (3)
C2—C3—C4—N2	−179.66 (19)	O2—C13—C14—C15	−180.0 (2)
C8—C3—C4—N2	0.0 (3)	C12—C13—C14—C15	0.3 (4)
C18—N2—C4—C5	93.5 (3)	C13—C14—C15—C16	0.4 (4)
C18—N2—C4—C3	−90.1 (3)	C14—C15—C16—C11	−0.4 (4)
C3—C4—C5—C6	5.2 (3)	C12—C11—C16—C15	−0.2 (3)
N2—C4—C5—C6	−178.49 (19)	C10—C11—C16—C15	178.8 (2)
C3—C4—C5—C9	−175.3 (2)	C4—N2—C18—C19	174.8 (2)
N2—C4—C5—C9	1.0 (3)	N2—C18—C19—C24	−3.0 (3)
C2—C1—C6—C5	−1.0 (3)	N2—C18—C19—C20	179.1 (2)
C7—C1—C6—C5	179.9 (2)	C24—C19—C20—C21	−0.2 (4)
C2—C1—C6—N1	173.84 (19)	C18—C19—C20—C21	177.7 (3)
C7—C1—C6—N1	−5.2 (3)	C19—C20—C21—C22	−0.8 (5)
C4—C5—C6—C1	−3.0 (3)	C20—C21—C22—C23	1.2 (5)
C9—C5—C6—C1	177.5 (2)	C25—O4—C23—C22	18.3 (4)
C4—C5—C6—N1	−178.06 (18)	C25—O4—C23—C24	−162.4 (3)
C9—C5—C6—N1	2.4 (3)	C21—C22—C23—O4	178.7 (3)
C10—N1—C6—C1	58.0 (3)	C21—C22—C23—C24	−0.5 (4)
C10—N1—C6—C5	−126.9 (2)	C20—C19—C24—O3	−179.0 (2)
C6—N1—C10—C11	−177.39 (19)	C18—C19—C24—O3	3.0 (3)
N1—C10—C11—C12	1.3 (3)	C20—C19—C24—C23	0.9 (3)
N1—C10—C11—C16	−177.7 (2)	C18—C19—C24—C23	−177.1 (2)
C16—C11—C12—O1	−179.3 (2)	O4—C23—C24—O3	0.1 (3)
C10—C11—C12—O1	1.7 (3)	C22—C23—C24—O3	179.4 (2)
C16—C11—C12—C13	0.8 (3)	O4—C23—C24—C19	−179.9 (2)
C10—C11—C12—C13	−178.2 (2)	C22—C23—C24—C19	−0.6 (4)

### Hydrogen-bond geometry (Å, º)

**Table:**

*D*—H···*A*	*D*—H	H···*A*	*D*···*A*	*D*—H···*A*
O1—H1···N1	0.89 (3)	1.76 (4)	2.566 (3)	150 (3)
O3—H3···N2	0.87 (4)	1.83 (4)	2.618 (2)	150 (4)
C26—H26···O1^i^	0.98	2.16	3.071 (4)	154

Symmetry code: (i) −*x*+1, −*y*+1, −*z*+1.

## References

[bb1] Allen, F. H., Kennard, O., Watson, D. G., Brammer, L., Orpen, A. G. & Taylor, R. (1987). *J. Chem. Soc. Perkin Trans. 2*, pp. S1–19.

[bb2] Bruker (2000). *SADABS*, *SMART* and *SAINT* Bruker AXS Inc., Madison, Wisconsin, USA.

[bb3] Hernández-Molina, R., Mederos, A., Gili, P., Domínguez, S., Lloret, F., Cano, J., Julve, M., Ruiz-Pérez, C. & Solans, X. (1997). *J. Chem. Soc. Dalton Trans.* pp. 4327–4334.

[bb4] Nardelli, M. (1995). *J. Appl. Cryst.* **28**, 659.

[bb5] Sheldrick, G. M. (2008). *Acta Cryst.* A**64**, 112–122. 10.1107/S0108767307043930 18156677

[bb6] Spek, A. L. (2009). *Acta Cryst.* D**65**, 148–155. 10.1107/S090744490804362X PMC2631630 19171970

[bb7] Yamin, B. M., Bakar, S. N. A., Kassim, K. & Bahron, H. (2009). *Acta Cryst.* E**65**, o2573. 10.1107/S1600536809037386 PMC2970302 21578010

